# Analysis of the differences between HPV-independent and HPV-related cervical adenocarcinoma

**DOI:** 10.3389/fonc.2025.1544207

**Published:** 2025-04-17

**Authors:** Jing He, Gulixian Tu lu Weng Jiang, Lin Zeng

**Affiliations:** ^1^ Department of Pathology, The Third Hospital of Mianyang, Sichuan Mental Health Center, Mianyang, China; ^2^ Department of Critical Care Medicine, Cancer Hospital Affiliated to Xinjiang Medical University, Urumqi, China

**Keywords:** cervical adenocarcinoma, HPV-related cervical adenocarcinoma, HPV-independent cervical adenocarcinoma, prognosis, retrospective cohort study

## Abstract

**Background:**

No prognostic evaluation criteria have been established for either human papillomavirus (HPV)-related cervical adenocarcinoma (HPV-CA) or HPV-independent cervical adenocarcinoma (HPV-Ind-CA). We aimed to compare and analyze the clinicopathological features and survival prognosis of patients with HPV-related and HPV-independent cervical adenocarcinoma to facilitate clinical diagnosis, treatment and survival prognosis of patients with cervical adenocarcinoma.

**Methods:**

Clinical data were collected from 47 patients with HPV-Ind-CA (HPV-Ind-CA group) and 285 patients with HPV-CA (HPV-CA group), who were diagnosed at the Oncology Hospital affiliated with Xinjiang Medical University between October 2012 and July 2023. A retrospective analysis was performed to compare the clinical characteristics (including age, ethnicity, fundamental diseases, initial symptoms, gynecological examination findings such as contact bleeding, menstrual history with regard to menopausal status, history of pregnancy and abortion, coexisting benign uterine lesions, etc.), tumor markers (CEA, CA125, CA199), HPV-infection(HPVI), treatment regimens (surgery, radiotherapy and chemotherapy), and pathological results (specific pathological subtype, maximum lesion diameter, degree of tumor differentiation, International Federation of Gynecology and Obstetrics [FIGO] stage) between the two groups. All patients were followed up until July 30, 2023 and differences in therapeutic efficacy (complete response, stable disease, progressive disease) and survival outcomes (progression-free survival [PFS] and overall survival [OS]) were compared between the two patient groups following treatment.

**Results:**

The proportions of patients in the HPV-Ind-CA and HPV-CA groups were 14.2% and 85.8%, respectively. Most patients presented with irregular vaginal bleeding, and a significant number experienced contact bleeding. The patients in the HPV-Ind-CA group were older (≥50 years), had a lower degree of differentiation, a later FIGO stage (> I stage), higher CA125 levels, and constituted a greater number of postmenopausal patients (P < 0.05) than those in the HPV-CA group. The median PFS and OS in the HPV-Ind-CA group were 12 ± 25.5 and 21 ± 25.1 months, respectively; the 3-year OS and PFS rates were 70.21%, and 59.57%, respectively. In contrast, the median PFS in the HPV-CA group was significantly longer at 26 ± 32.3 months, with a corresponding median OS of 35 ± 31.9 months; their respective 3-year OS and PFS rates were 80.70% and 73.68%. The HPV-Ind-CA group demonstrated significantly shorter PFS and OS than the HPV-CA group (P < 0.05). In the HPV-Ind-CA cohort, low cervical adenocarcinoma differentiation (HR=152.673, 95% CI: 1.777–13117.314, P=0.027) and high CA199 levels (HR=104.888, 95%CI: 2.420–4546.373, P=0.016) were independent prognostic factors for OS. Conversely, in the HPV-CA cohort, the FIGO stage (> stage I), absence of HPVI, and high CA125 levels were independent prognostic factors influencing both OS and PFS outcomes; additionally, older age ≥ 50 years and high CA199 levels were independent risk factors for OS and PFS, respectively. Thus, greater than stage I FIGO stage and high CA125 and CA199 levels were identified as independent prognostic factors for PFS and OS in cervical adenocarcinoma. Furthermore, the absence of HPVI and older age of ≥50 years also constituted as independent risk factors for OS in this patient population.

**Conclusions:**

Compared with HPV-CA, HPV-Ind-CA is associated with inferior clinicopathological characteristics and survival outcomes. For patients who are HPV-negative and have elevated levels of CA125 and CA199, with a FIGO stage of I or higher, it is advisable to contemplate an intensified treatment protocol. This approach aims to enhance the opportunity for curative surgical resection when the patient’s physical condition permits it, thereby improving the overall prognosis.

## 1 Introduction

Cervical cancer is one of the most prevalent gynecological malignancies and the fourth most common neoplasm worldwide. In 2018, approximately 570,000 new cancer cases were reported worldwide, accounting for 6.6% of all cancer cases diagnosed in women (1). In developing countries, the incidence and mortality of cervical cancer occupy the second position; whereas in developed countries, it ranks the tenth (2). Epidemiological studies have demonstrated that the incidence and mortality rates of cervical cancer in China are among the highest globally, imposing a substantial disease burden on the population (3, 4). The most prevalent type is cervical squamous cell carcinoma (SCC) associated with human papillomavirus (HPV)-infection (HPVI) (5). Adenocarcinomas account for approximately 25% of cervical cancer cases, of which approximately 15% are unrelated to HPV (6). The prevalence of HPVI in patients with invasive cervical cancer ranges from 85% to 99%. High-risk HPV types 16 and 18 comprise approximately 70–80% of SCC, whereas HPV types 16, 18, and 45 account for approximately 94% of cervical adenocarcinomas (7). In recent years, with the widespread implementation of cervical cancer screening programs, the incidence of squamous epithelial lesions has consistently decreased, whereas that of glandular epithelial lesions has continuously increased (8). In 2020, the fifth edition of the World Health Organization revised the classification of cervical adenocarcinoma based on the International Endocervical Adenocarcinoma Criteria and Classification (IECC) and its association with HPV. Cervical adenocarcinomas are categorized into two primary types: HPV-related and HPV-independent (9). Usual type and mucinous cervical adenocarcinoma (specifically the intestinal type, signet ring cell type) is the most commonly encountered type of HPV-related cervical adenocarcinoma (HPV-CA). Other subtypes of HPV-CA encompass invasive stratified mucinous carcinoma (ISMC) as well as non-specific mucinous adenocarcinoma. HPV-Independent cervical adenocarcinoma (HPV-Ind-CA) includes gastric-type adenocarcinoma (G-EAC), clear-cell carcinoma, mesonephric adenocarcinoma, endometrioid adenocarcinoma, and non-specific adenocarcinoma (8, 9).

Currently, research on cervical adenocarcinoma represents a significant area of interest, as existing guidelines do not differentiate treatment approaches for these lesions from those applicable to squamous lesions. However, increasing evidence suggests that the biological behavior, therapeutic outcomes, and prognostic factors associated with cervical adenocarcinoma are distinct from those associated with SCC. Furthermore, cervical adenocarcinomas exhibit reduced sensitivity to radiotherapy and chemotherapy and are associated with poorer prognoses than SCC (10). Moreover, to date, no prognostic evaluation criteria have been established for HPV-CA or HPV-Ind-CA globally. Consequently, an examination of the differences in the clinical and pathological characteristics between patients with HPV-Ind-CA and those with HPV-CA is of considerable significance for clinical diagnosis and treatment outcomes.

This study aimed to comparatively analyze the clinicopathological characteristics, therapeutic efficacy assessments, and survival prognoses of the HPV-CA and HPV-Ind-CA groups to further investigate the key factors influencing prognosis, recurrence, and metastasis with the aim of providing valuable insights into the diagnosis and treatment of such patients in clinical practice.

## 2 Materials and methods

### 2.1 Study population

Data were collected from 332 patients diagnosed (through radical surgery/biopsy/resection specimens) with cervical adenocarcinoma at the Oncology Hospital affiliated with Xinjiang Medical University between October 2012 and July 2023 and categorized into the HPV-Ind-CA and HPV-CA groups. Diagnosis was established in accordance with the World Health Organization classification criteria for adenocarcinoma published in 2020. The HPV-CA group included well-differentiated adenocarcinoma and mucinous adenocarcinoma (intestinal type, ISMC, and nonspecific mucinous adenocarcinoma), whereas the HPV-Ind -CA group included G-EAC, clear cell carcinoma, endometrioid adenocarcinoma, and nonspecific invasive adenocarcinoma. The HPV-Ind-CA group comprised 14.2% (47/332) and the HPV-CA group comprised 85.8% (285/332) of the participants. A retrospective comparative analysis was conducted to evaluate differences in clinicopathological characteristics, auxiliary examination results, treatment modalities, prognostic outcomes, and survival rates between the two groups.

### 2.2 Monitoring of survival

All patients were followed up through outpatient consultations and telephone communication. Among the participants, four were lost to follow-up, resulting in an overall follow-up rate of 97.32%. The follow-up duration varied from 2 to 217 months, with a median of 37 months. Statistical analysis of therapeutic outcomes included complete remission (CR), stable disease (SD), disease progression (PD), and mortality. We evaluated overall survival (OS) from diagnosis until patient death and progression-free survival (PFS) from diagnosis to PD.

### 2.3 Statistical analysis

Data were analyzed utilizing SPSS 26.0 statistical software. Categorical data were subjected to the chi-square test and are expressed as percentages (%). Survival curves were evaluated using the Kaplan–Meier method, and differences in PFS and OS between the two groups were compared using log-rank regression analysis. The Cox proportional hazards model was used for prognostic factor analysis. Statistical significance was set at P < 0.05.

## 3 Results

### 3.1 Basic clinical characteristics

The age at diagnosis of the 332 patients with cervical adenocarcinoma ranged from 27 to 82 years, with a mean age of 52 years. There was no significant disparity between the two groups of patients regarding underlying diseases, such as heart disorders, vascular disorders and diabetes mellitus (**Table 1**). The predominant concern among the patients with cervical adenocarcinoma at the time of medical consultation was abnormal vaginal bleeding and discharge (71.7%, 238/332). Additionally, 65.4% (217/332) of patients experienced contact bleeding (**Table 1**). The overall HPVI rate among patients was 65.36% (217/332), with 10.6% (5/47) in the HPV-Ind-CA group and 74.4% (212/285) in the HPV-CA group. The median age at diagnosis of patients with cervical adenocarcinoma was 56 years in the HPV-Ind-CA group and 51 years in the HPV-CA group. Statistical analysis indicated a significant difference between the two groups (χ^2^ = 11.166, P = 0.001). The maximum tumor diameter ranged from 0.5 to 12.9 cm, with a mean of 4.6 cm. Among them, 70.2% (233/332) underwent radical hysterectomy for cervical cancer. Excluding age as a variable, statistically significant differences were observed between the HPV-Ind-CA and HPV-CA groups regarding menstrual history (menopausal status), FIGO stage, variations in tumor markers (CA125), performance of radical surgery, and degree of differentiation (P< 0.05). The HPV-Ind-CA group exhibited older age, greater FIGO stage, lower degree of differentiation, higher CA125 levels, and a higher proportion of postmenopausal patients, as well as those not suitable for radical surgery, than the HPV-CA group (P < 0.05). Conversely, no statistically significant differences were observed between the two groups in terms of ethnicity, initial symptoms, presence of contact bleeding, coexisting benign uterine lesions (adenomyosis, leiomyoma, and ectopic pregnancy), variations in tumor markers (CA199 and CEA), treatment modalities (radiotherapy and chemotherapy), presence of HPVI, or maximum tumor diameter (P > 0.05; **Tables 1**–**3**).

**Table 1:** Basic clinical characteristics of patients diagnosed with cervical adenocarcinoma in the two groups.

Basic clinical characteristics	HPV-Ind-CA group	HPV-CA group	χ^2^/fisher	P
Age, years				
≥50	37	150	11.166	0.001^*^
<50	10	135		
Asian ethnicity				
Han	32	201	7.673	0.362
Hui	6	12		
Uigur	9	59		
Manchu	0	3		
Kirgiz	0	1		
Kazak	0	6		
Mongolian	0	2		
Dongxiang	0	1		
Symptoms				
Abnormal vaginal hemorrhage	28	164	3.034	0.882
Abnormal vaginal secretion	7	39		
Other	1	20		
None	13	89		
Vascular disorders				
Yes	9	48		
No	36	226	0.162	0.687
Cardiac disorders				
Yes	9	41		
No	36	233	0.742	0.389
Diabetes mellitus				
Yes	7	19		
No	38	255	3.838	0.050
Parity				
<2	11	38	3.391	0.066
≥2	35	244		
Miscarriage incidence				
<2	30	174	0.234	0.629
≥2	16	109		
Menopause				
Yes	33	150	5.738	0.017^*^
No	13	134		
Bleeding^△^				
Yes	27	190	1.515	0.218
No	20	95		
Correlation with other benign uterine lesions				
Adenomyoma	3	19	5.958	0.202
Leiomyoma	9	96		
Ectopic gestation	0	2		
No	37	178		
HPVI				
Yes	5	212		
No	42	73	-	-

HPV-CA, human papillomavirus-related cervical adenocarcinoma; HPVI, human papillomavirus infection; HPV-Ind-CA, human papillomavirus-independent cervical adenocarcinoma; ^△^denotes the occurrence of contact hemorrhage. ^*^P<0.05.

**Table 2:** A comparative analysis of the alterations in tumor markers between the two patient groups.

Index	HPV-Ind-CA group	HPV-CA group	χ^2^/fisher	P
CA125				
Increase	25	110	4.477	0.034^*^
Normal	19	166		
CA199				
Increase	16	68	2.387	0.122
Normal	29	208		
CEA				
Increase	12	71	0.006	0.936
Normal	33	201		

HPV-CA, human papillomavirus-related cervical adenocarcinoma; HPV-Ind-CA, human papillomavirus-independent cervical adenocarcinoma; ^*^P<0.05.

**Table 3:** A comparative analysis of the treatment protocols between the two patient groups.

Clinical Treatment Protocols	HPV-Ind-CA group	HPV-CA group	χ^2^/fisher	P
Surgery				
Yes	22	211	14.955	0.000^*^
No	25	72		
Adjuvant radiotherapy				
Yes	35	177	5.350	0.069
No	11	107		
Adjuvant chemotherapy				
Yes	32	188	0.014	0.800
No	15	96		

HPV-CA, human papillomavirus-related cervical adenocarcinoma; HPV-Ind-CA, human papillomavirus-independent cervical adenocarcinoma; ^*^P<0.05.

### 3.2 Pathological characteristics

The predominant pathological type was usual type cervical adenocarcinoma, comprising 76.8% (255/332) of cases. Other subtypes of HPV-CA included 20 (6.02%) of mucinous adenocarcinoma, not otherwise specified(NOS)、9 (2.71%) of intestinal-type mucinous adenocarcinoma and 1 (0.30%) of ISMC. Within the HPV-Ind-CA group, the predominant subtype was gastric-type adenocarcinoma, which included three cases of micro invasive adenocarcinoma and constituted 9.04% of all cases. Other subtypes of HPV-Ind-CA included 7 (2.11%) of invasive adenocarcinoma, NOS、6 cases (1.81%) of endometrioid adenocarcinoma and 4 (1.20%) of clear cell carcinoma. A total of 65.4% (217/332) of patients tested positive for HPV, with only 31.63% (153/178) classified as having FIGO stage I. Most patients exhibited cervical masses, and 75.30% (250/332) had tumors with a maximum diameter of at least 2 cm. The distribution of patients by grade of cervical adenocarcinoma was as follows: high-grade, 11.45%; moderate-grade, 64.46%; and low-grade, 24.10% (**Table 4**).

**Table 4:** A comparative analysis of the pathological characteristics of patients with cervical adenocarcinoma in the two groups.

Pathological characteristics	HPV-Ind-CA group	HPV-CA group	χ^2^/fisher	P值
Diagnosis				
Usual type		255	–	–
Intestinal-type		9		
G-EAC	30			
Endometrioid adenocarcinoma	6			
Clear cell type	4			
ISMC		1		
Mucinous adenocarcinoma, NOS		20		
Invasive adenocarcinoma, NOS	7			
HPVI				
Yes	5	212	–	–
No	42	73		
MD^▲^(cm)				
<2	2	19	0.871	0.647
≥2, <4	13	66		
≥4	30	141		
Differentiation				
High	12	26		
Moderate	18	196	18.617	0.000^*^
Low	17	63		
FIGO stage				
I	7	98	20.699	
II	15	121		
III	14	42		0.000^*^
IV	8	14		

FIGO, International Federation of Gynecology and Obstetrics; G-EAC, gastric-type adenocarcinoma; HPV-CA, human papillomavirus-related cervical adenocarcinoma; HPVI, human papillomavirus infection; ISMC, invasive stratified mucinous carcinoma; HPV-Ind-CA, human papillomavirus-independent cervical adenocarcinoma; MD, maximum diameter; NOS, not otherwise specified; ^▲^ denotes the largest dimension of the neoplasm. ^*^P<0.05.

### 3.3 Assessment of treatment efficacy and survival analysis

**Figure 1:**
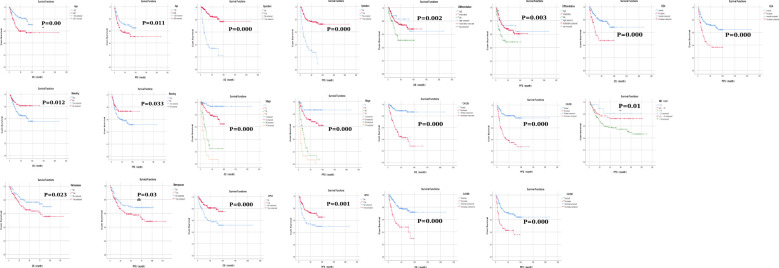
Kaplan–Meier curves of factors affecting progression-free survival and overall survival in patients with cervical adenocarcinoma.

No significant differences were observed in the efficacy assessment (CR, SD, and PD) between the two patient groups (P > 0.05), and there was no statistically significant difference in the proportion of patients who succumbed to tumors in either group (**Table 5**). The log-rank univariate analysis indicated that factors such as older age of ≥50 years, presence of contact bleeding, postmenopausal status, absence of HPVI, high levels of serum tumor markers (CA125, CA199, CEA), lack of radical surgery, FIGO stage > I, and low differentiation were identified as risk factors for OS and PFS in patients with cervical adenocarcinoma. Furthermore, patients with a maximum tumor diameter ≥2 cm exhibited significantly shorter PFS (P < 0.05), as detailed in **Table 6** and **Figure 1**.

**Table 5:** A Comparative analysis of therapeutic efficacy and survival outcomes of patients with cervical adenocarcinoma in the two groups.

Evaluation	CR	SD	PD	Dead	Lost to follow up	PFS (month)	OS (month)
HPV-CA	53	143	85	67	4	26 ± 32.34	35 ± 31.93
HPV-Ind-CA	11	17	19	15	0	12 ± 25.48	21 ± 25.11
χ^2^	4.233			1.399		5.895	4.695
P value	0.237			0.237		0.015^*^	0.030^*^

CR, complete remission; HPV-CA, human papillomavirus-related cervical adenocarcinoma; HPV-Ind-CA, human papillomavirus-independent cervical adenocarcinoma; OS, overall survival; PD, disease progression; PFS, progression-free survival; SD, stable disease; ^*^P<0.05.

**Table 6:** A summary of the univariate log-rank survival analysis for the two patient groups (χ^2^).

Factors	PFS		OS	
	χ^2^	P value	χ^2^	P value
Age, y				
<50				
≥50	6.435	0.011^*^	11.74	0.001^*^
Asian ethnicity				
Han				
Hui	3.531	0.832	4.910	0.671
Uigur				
Manchu				
Kirgiz				
Kazak				
Mongolian				
Dongxiang				
Symptoms				
Abnormal vaginal hemorrhage				
Abnormal vaginal secretion	7.982	0.334	8.409	0.298
Other				
None				
Bleeding				
No				
Yes	4.554	0.033^*^	6.313	0.012^*^
Menopause				
No				
Yes	4.716	0.030^*^	5.189	0.023^*^
Correlation with other benign uterine lesions				
Adenomyoma				
Leiomyoma	2.252	0.690	1.594	0.810
Ectopic gestation				
No				
Miscarriage incidence				
<2				
≥2	3.247	0.072	2.288	0.130
Parity				
<2				
≥2	0.000	0.994	0.283	0.595
Differentiation				
High				
Moderate	11.389	0.003^*^	12.194	0.002^*^
Low				
HPVI				
No				
Yes	10.918	0.001^*^	15.515	0.000^*^
MD (cm)				
<2				
≥2, <4	7.879	0.019^*^	5.315	0.070
≥4				
FIGO stage				
I				
II	147.892	0.000^*^	159.883	0.000^*^
III				
IV				
CA125				
Normal				
Increase	59.585	0.000^*^	67.180	0.000^*^
CA199				
Normal				
Increase	26.929	0.000^*^	31.872	0.000^*^
CEA				
Normal				
Increase	21.546	0.000^*^	20.722	0.000^*^
Surgery				
Yes				
No	75.555	0.000^*^	99.741	0.000^*^
Adjuvant radiotherapy				
No				
Yes	1.973	0.160	5.801	0.055
Adjuvant chemotherapy				
No				
Yes	0.039	0.843	2.952	0.086

HPV-CA, human papillomavirus-related cervical adenocarcinoma; HPVI, human papillomavirus infection; HPV-Ind-CA, human papillomavirus-independent cervical adenocarcinoma; OS, overall survival; PFS, progression-free survival; ^*^P < 0.05.

In summary, multivariate Cox regression analysis revealed that FIGO stage > I and high levels of CA125 and CA199 were independent risk factors for PFS and OS in patients with cervical adenocarcinoma. Furthermore, the absence of HPVI and older age of ≥ 50 years were also recognized as independent risk factors for OS, all exhibiting statistically significant differences (P < 0.05) (**Table 7**). Among these patients, low differentiation (hazard ratio [HR] = 152.673, 95% confidence interval [CI]: 1.777–13117.314, P = 0.027) and high CA199 levels (HR = 104.888, 95% CI: 2.420–4546.373, P = 0.016) were identified as significant independent risk factors for OS in those with HPV-Ind-CA (P < 0.05) (**Table 8**). In patients with HPV-CA, FIGO stage > I, absence of HPVI, and high CA125 levels were identified as independent risk factors influencing OS and PFS. Furthermore, older age ≥50 years was recognized as an significant independent risk factor affecting OS and high CA199 level affecting PFS (P < 0.05) (**Table 9**).

**Table 7:** Multivariate Cox regression analysis of prognostic factors in patients with cervical adenocarcinoma [HR(95%CI)].

Factor	PFS	P value	OS	P value
Age(≥ 50 years)	1.421(0.808–2.497)	0.222	02.538(1.309–4.921)	0.006^*^
FIGO stage (> I)	1.857(1.251–2.756)	0.002^*^	2.3329(1.511–3.598)	0.000^*^
HPVI (No)	0.635(0.380–1.061)	0.083	0.474(0.256–0.877)	0.017^*^
CA125 (increase)	2.163(1.188–3.937)	0.012^*^	2.560(1.285–5.097)	0.007^*^
CA199 (increase)	2.294(1.335–3.944)	0.003^*^	2.326(1.266–4.275)	0.007^*^

FIGO, International Federation of Gynecology and Obstetrics; HPVI, human papillomavirus infection; OS, overall survival; PFS, progression-free survival; ^*^P<0.05.

**Table 8:** Multivariate Cox survival analysis of prognostic factors in patients with HPV-Ind-CA [HR 95%CI)].

Factor	PFS	P value	OS	P value
Differentiation (Low)	1.754 (0.614–5.015)	0.294	152.673 (1.777–13117.314)	0.027^*^
CA199 (Increase)	2.865 (0.575–14.276)	0.199	104.888 (2.420–4546.373)	0.016^*^

HPV-Ind-CA, human papillomavirus-independent cervical adenocarcinoma; OS, overall survival; PFS, progression-free survival; ^*^P<0.05.

**Table 9:** Multivariable Cox survival analysis of prognostic factors in patients with HPV-CA [HR(95%CI)].

Factor	PFS	P value	OS	P value
Age(≥ 50 years)	1.685(0.908–3.127)	0.098	2.614(1.304–5.242)	0.007^*^
FIGO stage(> I)	1.689(1.082–2.636)	0.021^*^	2.381(1.513–3.747)	0.000^*^
HPVI(No)	0.566(0.323–0.991)	0.047^*^	0.443(0.231–0.851)	0.014^*^
CA125 (increase)	2.407(1.280–4.529)	0.006^*^	2.835(1.371–5.862)	0.005^*^
CA199 (increase)	2.216(1.191–4.123)	0.012^*^	-	-

FIGO, International Federation of Gynecology and Obstetrics; HPV-CA, human papillomavirus-related cervical adenocarcinoma; HPVI, human papillomavirus infection; OS, overall survival; PFS, progression-free survival; ^*^P<0.05.

**Figure 2:**
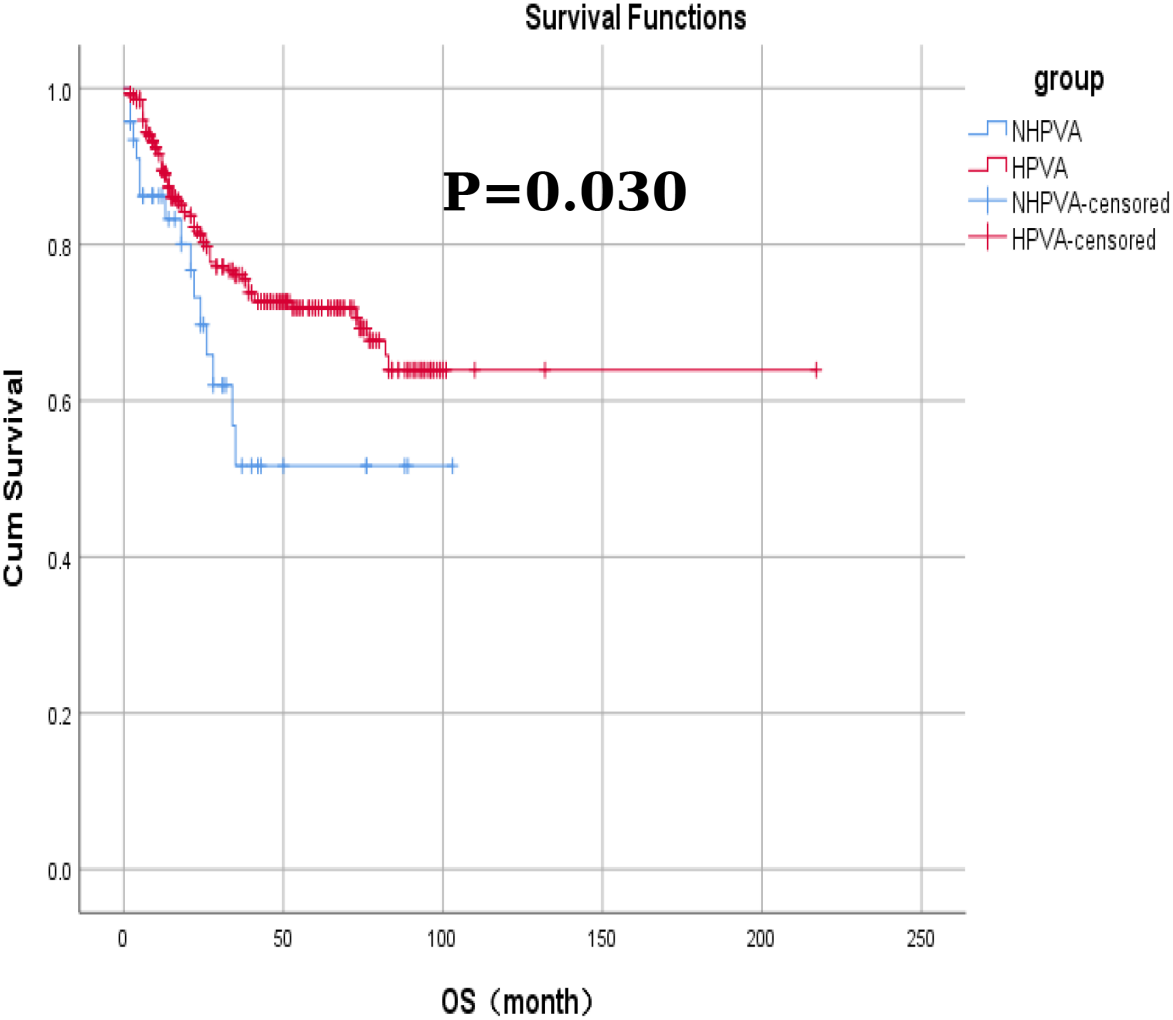
The Kaplan–Meier curves for overall survival in patients in the HPV-Ind -CA and HPV-CA groups.

**Figure 3:**
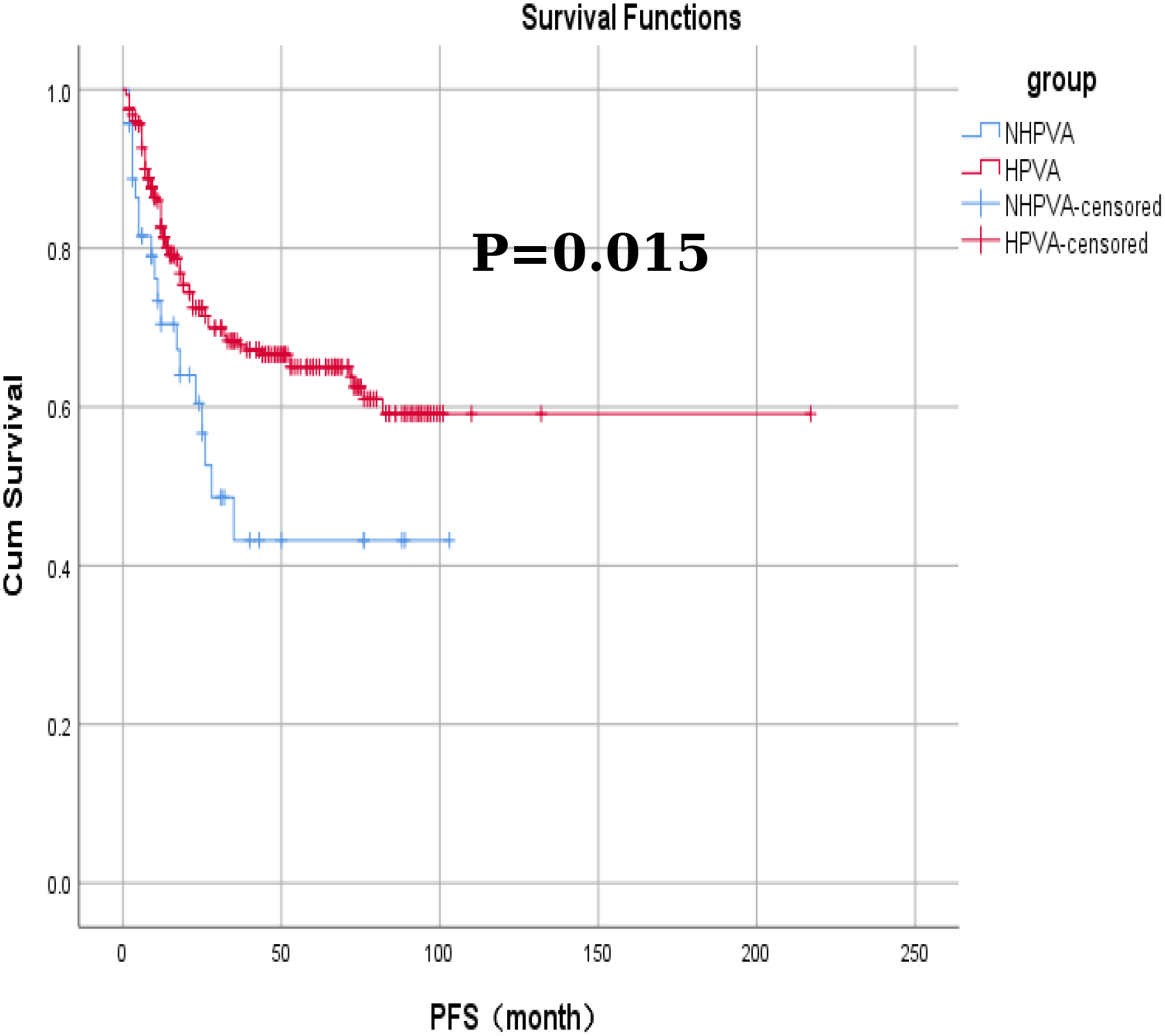
The Kaplan-Meier curves for progression-free survival in patients in the HPV-Ind -CA and HPV-CA groups.

Among the 332 patients, the OS rate was 74.1% (246/332) months in the HPV-Ind-CA group, with the mean PFS and OS of 12 ± 25.5 and 21 ± 25.1 months, respectively. Survival analysis revealed that the 3-year OS and PFS rates were 70.21% and 59.57%, respectively. In the HPV-CA group, the mean PFS and OS were 26 ± 32.3 and 35 ± 31.9 months, respectively. Survival analysis revealed that the 3-year OS and PFS rates were 80.70% and 73.68%, respectively. Statistically significant differences in OS and PFS were observed between the two groups of patients with cervical adenocarcinoma. The HPV-CA group demonstrated superior OS (χ^2^ = 4.695, P = 0.030) and PFS (χ^2^ = 5.895, P = 0.015) compared to the HPV-Ind-CA group. Detailed information can be found in **Figures 2**, **3**.

## 4 Discussion

With the widespread implementation of cervical cancer screening methods and introduction of cervical cancer vaccines, the incidence of cervical SCC has significantly declined. Compared to cervical squamous epithelial lesions, liquid-based cytology demonstrates a lower detection rate for cervical glandular epithelial lesions. This discrepancy arises from the deeper location of the glandular epithelial lesions within the cervix. Consequently, when collecting samples for cervical cytology, relying solely on surface cell collection may lead to an increased risk of misdiagnosis (11, 12). Consequently, HPV screening and cervical liquid-based cytological assessments have limited utility in the early detection of cervical adenocarcinoma (13). Physicians with inadequate awareness of rare diseases may be unable to identify such conditions promptly during gynecological examinations. Consequently, early diagnosis and intervention are often not realized, contributing to the rising incidence of cervical adenocarcinoma.

The etiology of cervical adenocarcinoma remains unclear. Similar to cervical SCC, it may be associated with several factors as follows: 1) persistent high-risk HPVIs (particularly types 16 and 18); 2) epigenetic alterations, genetic mutations, and dysregulation of the cell cycle; 3) prolonged chronic inflammation leading to changes in the microenvironment that facilitate tumor development and progression; 4) early onset of sexual activity, disordered sexual behaviors among partners, multiple sexual partners, and excessive reproductive history; and 5) obesity, prolonged use of oral contraceptives (exceeding 10 years), and hormone replacement therapy (14, 15). The onset of cervical adenocarcinoma is typically insidious and its clinical manifestations lack specificity. The mean age of the patients at diagnosis is 50 years. Notably, patients with HPV-Ind-CA tend to be older than those with HPV-CA, frequently presenting in the elderly population (16). Irregular vaginal bleeding often serves as the initial symptom prompting medical consultation (17). The current study revealed that the mean age of the patients diagnosed with cervical adenocarcinoma was 52 years. The majority of patients with HPV-Ind-CA were aged ≥ 50 years (P = 0.001), and most were postmenopausal (P = 0.023), indicating a statistically significant difference from those in the HPV-CA group. Furthermore, 57.83% of patients presented with abnormal vaginal bleeding as their initial symptom, and a substantial proportion exhibited contact bleeding during gynecological examinations, which is consistent with findings from previous studies. In patients with cervical adenocarcinoma, gynecological examinations often reveal cervical hypertrophy and endophytic growth of lesions, which typically have a barrel-shaped appearance. Cervical polyps may also be observed in some patients (18). These lesions predominantly originate from the deep cervical stroma and demonstrate endophytic growth, making them susceptible to misdiagnosis or missed diagnoses before surgery. Their biological behavior is notably more aggressive; compared to SCC, they are more likely to metastasize to the pelvic lymph nodes, invade stromal tissue, and infiltrate deeper into the cervix (19).

Treatment strategies for cervical adenocarcinoma are analogous to those for cervical SCC, and no standardized therapeutic approach is currently available. The 2018 IECC established that HPV-Ind-CA possesses distinct morphological and molecular characteristics associated with poor prognosis and varying potential for future targeted therapies (20). The current study demonstrated that the absence of radical surgery is a significant risk factor for PFS and OS in patients with cervical adenocarcinoma. Therefore, for postmenopausal patients with HPV-Ind-CA with FIGO stage > I, neoadjuvant chemoradiotherapy is a viable option that may enhance the likelihood of surgical resection if the patient’s physical condition allows it. In addition to the standard radical hysterectomy and lymphadenectomy, concurrent excision of the greater omentum, appendix, and metastatic lesions is recommended. Postoperatively, concurrent chemoradiotherapy with platinum agents can be augmented with immunosuppressants to improve patient prognosis.

Using the IECC classification, the case data of patients with cervical adenocarcinoma collected in this study were categorized into HPV-Ind-CA and HPV-CA groups for a comparative analysis. The results revealed statistically significant differences between the two groups in several parameters, including age (median age, 56 and 51 years, respectively), menstrual history (menopausal status), degree of differentiation, FIGO staging, variations in the tumor marker CA125, and whether radical surgery was performed (P < 0.05). The HPV-Ind-CA group exhibited greater FIGO staging (P = 0.000), lower tumor differentiation (P = 0.000), and higher CA125 levels (P = 0.034). Additionally, most patients in this group were postmenopausal (P = 0.017) and largely unable to undergo radical surgery (P = 0.000). The tumor-related mortality rate was higher in the HPV-Ind-CA group (31.91%, 15/47) than in the HPV-CA group (23.51%, 67/285); however, this difference was not statistically significant (P > 0.05). Furthermore, the findings indicated that OS and PFS were significantly poorer in the HPV-Ind-CA group (χ^2^ = 4.695, P = 0.030 and χ^2^ = 5.895, P = 0.015, respectively) than in the HPV-CA group, which aligns with previous research findings.

In recent years, a growing body of literature on cervical adenocarcinoma has indicated that the factors influencing its prognosis include age, HPV infection subtype, clinical stage, histological subtype, maximum tumor diameter, stromal invasion, presence of a vascular tumor thrombus, surgical resection margin status, and ovarian and lymph node metastases. However, no definitive conclusion has been reached. Lijie et al. (21) conducted a study examined the correlation between HPVI subtypes, viral load, and the clinicopathological characteristics of cervical adenocarcinoma and reported that HPV-Ind-CA was associated with either an absence of viral infection or a lower viral load than HPV-CA. Furthermore, low viral loads observed in cases of multiple infections were identified as significant factors contributing to poor survival prognosis. We conducted a log-rank univariate survival analysis to assess HPVI status in the two patient groups. The results revealed that the absence of HPVI constituted an independent risk factor for OS in cervical adenocarcinoma (HR = 0.474, 95% CI: 0.256 - 0.877, P = 0.017), thereby further corroborating the aforementioned research findings. Previous studies have indicated that HPV-Ind-CA is diagnosed at a more advanced stage and is associated with higher rates of lymph node metastasis, reduced PFS and OS, and poorer prognosis (22). G-EAC, the most prevalent subtype of HPV-Ind-CA, is more aggressive than usual type cervical adenocarcinoma. More than 40% of patients are diagnosed with G-EAC at an intermediate or advanced stage of the disease (23). This could potentially be attributed to the reduced predictive capacity of negative HPV tests for cervical cancer, thereby resulting in the failure of timely detection of the disease at the early stage (24). The study further revealed that the 5-year survival rate for common cervical adenocarcinoma reached 91%, whereas that for G-EAC was only 42%. In addition, a higher FIGO stage was associated with poorer PFS and OS. The current study identified FIGO stage beyond Stage I as an independent risk factor for PFS and OS in patients with cervical adenocarcinoma, thereby corroborating previous findings.

Currently, research investigating the correlation between tumor markers and cervical adenocarcinoma has increased significantly, with particular emphasis on CA-125 and CA19-9. Although these markers have traditionally been regarded as important indicators for ovarian epithelial tumors, their roles in cervical adenocarcinoma are now receiving increasing attention. Previous studies indicated that approximately half of the patients with G-EAC exhibited high CA199 levels, and approximately one-third had high CA125 levels, and an increase in CA125 is often indicative of pelvic and abdominal metastasis (25). Once again, Jack et al. (26) conducted a retrospective analysis of the clinical data from 572 patients with International Federation of FIGO stage IA-IIA cervical adenocarcinoma and found that Stratified analysis showed that elevated CA125 and CA199 were significantly associated with postoperative distant metastasis. And a decision curve analysis confirmed that a combination of tumor markers as predictors significantly outperformed the other common predictors used (FIGO-stage, intermediate and high-risk factors, tumor differentiation, lymph nodes). The aforementioned research outcomes unanimously demonstrate that a significant correlation exists between the tumor markers CA125 and CA199 and the diagnosis and prognosis of cervical adenocarcinoma. In the comparative analysis of tumor markers between the two groups, we observed that a higher proportion of patients in the HPV-Ind-CA group exhibited high CA125 levels than those in the HPV-CA group. Additionally, high CA125 and CA199 levels were identified as independent risk factors for PFS and OS in cervical adenocarcinoma, suggesting that increased serum levels of CA125 and CA199 may serve as prognostic biomarkers for poor outcomes in this condition (27), and the findings of this research might offer significant evidence in support of the application of preoperative serum CA-125 and CA19-9 detection in the prognosis evaluation of cervical adenocarcinoma. however, further validation is necessary through studies involving larger sample sizes.

A major limitation of this study was the relatively limited sample size. Currently, there is a paucity of research on HPV-Ind-CA, globally, and the existing literature is insufficient. Further large-scale multicenter studies are necessary to comprehensively investigate the clinicopathological characteristics and prognostic differences between HPV-Ind-CA and HPV-CA, as well as to enhance awareness regarding cervical adenocarcinoma. Early identification, diagnosis, and treatment strategies are essential for improving the survival outcomes of patients with cervical adenocarcinoma.

Cervical adenocarcinoma is characterized by concealed anatomical location and deep-seated lesions, accompanied by atypical clinical symptoms and signs, which complicate its early detection. The findings of this study suggest that clinicians must consider seeking cervical glandular epithelial lesions when patients present with abnormal vaginal bleeding or discharge. During gynecological examinations, meticulous attention should be paid to the morphology of the cervix to assess characteristics, such as barrel-shaped enlargement, thickening, hypertrophy, or increased firmness. If indicated, enhanced magnetic resonance imaging (28), multiple colposcopic deep biopsies (29), or cervical conization may be used to elucidate the nature of the lesions for timely identification and diagnosis. For patients with a confirmed diagnosis of cervical adenocarcinoma without HPV infection, who are aged ≥50 years and have FIGO staging > I along with high CA125 and CA199 levels, it is imperative to implement standardized treatment protocols and conduct regular follow-up assessments to optimize survival outcomes.

In conclusion, compared with HPV-CA, HPV-Ind -CA is a heterogeneous tumor characterized by distinct clinical and molecular mechanisms. Owing to its rarity and negative HPV test results, it lacks specific clinical manifestations, is often overlooked by clinicians, complicates early diagnosis, has a high rate of misdiagnosis, is susceptible to invasion and metastasis, demonstrates insensitivity to radiotherapy and chemotherapy, and has a poor prognosis. However, there are currently no tailored treatment protocols for cervical adenocarcinoma that consider the HPV status or various histological subtypes in clinical practice. Understanding the clinicopathological features of patients with cervical adenocarcinoma who present with these risk factors will significantly aid in early detection, diagnosis, and management of this type of tumor. Additionally, efforts should be made to maximize surgical opportunities for patients to improve their survival outcomes.

## Data Availability

The original contributions presented in the study are included in the article/supplementary material. Further inquiries can be directed to the corresponding author.
