# Corrigendum: Analysis of the differences between HPV-independent and HPV-related cervical adenocarcinoma

**DOI:** 10.3389/fonc.2025.1613729

**Published:** 2025-05-09

**Authors:** Jing He, Gulixian Tu lu Weng Jiang, Lin Zeng

**Affiliations:** ^1^ Department of Pathology, The Third Hospital of Mianyang, Sichuan Mental Health Center, Mianyang, China; ^2^ Department of Pathology, Department of Critical Care Medicine, Cancer Hospital Affiliated to Xinjiang Medical University, Urumqi, China

**Keywords:** cervical adenocarcinoma, HPV-related cervical adenocarcinoma, HPV-independent cervical adenocarcinoma, prognosis, retrospective cohort study

In the published article, there was an error in affiliation(s) 1. Instead of “The Third Hospital of Mianyang, Mianyang, China”, it should be “The Third Hospital of Mianyang, Sichuan Mental Health Center, Mianyang, China”.

The authors apologize for this error and state that this does not change the scientific conclusions of the article in any way. The original article has been updated.

